# Novel InDels of *GHR*, *GHRH*, *GHRHR* and Their Association with Growth Traits in Seven Chinese Sheep Breeds

**DOI:** 10.3390/ani10101883

**Published:** 2020-10-15

**Authors:** Mingli Wu, Haidong Zhao, Xiaoqin Tang, Qi Li, Xiaohua Yi, Shirong Liu, Xiuzhu Sun

**Affiliations:** 1 College of Animal Science and Technology, Northwest A&F University, Yangling 712100, China; wumingli@nwafu.edu.cn (M.W.); 2018060160@nwafu.edu.cn (H.Z.); txq@nwafu.edu.cn (X.T.); liqi990@nwafu.edu.cn (Q.L.); yixiaohua@nwafu.edu.cn (X.Y.); liushirong221@nwafu.edu.cn (S.L.); 2 College of Grassland Agriculture, Northwest A&F University, Yangling 712100, China

**Keywords:** Chinese mutton sheep, growth and development, *GHR*, *GHRH*, *GHRHR*, InDels

## Abstract

**Simple Summary:**

It is advantageous to find the potential molecular markers which are associated with the growth and development of mutton sheep through molecular breeding. In this study, four molecular markers were selected from *growth hormone receptor* (*GHR*), one was found on *growth hormone releasing hormone* (*GHRH*) and *growth hormone releasing hormone receptor* (*GHRHR*), respectively. The purposes of this paper were to screen molecular makers in the related genes of the growth hormone (GH) axis and provide theoretical basis for molecular breeding and genetic selection of mutton sheep. In this study, six molecular markers were selected from the genes for *growth hormone receptor* (*GHR*), *growth hormone releasing hormone* (*GHRH*), and *growth hormone releasing hormone receptor* (*GHRHR*). The three molecular markers in *GHR* and one in *GHRHR* could potentially be used for marker assisted selection of growing-related traits in mutton sheep.

**Abstract:**

The GH growth axis plays an important role in the growth and development of animals and runs through the whole life of animals. Many studies have shown that molecular mutations in key genes of the GH axis will affect the growth and development of animals. The purpose of this study was to explore the distribution characteristics of InDels of *GHR*, *GHRH*, and *GHRHR* in seven Chinese sheep populations, and to further explore the relationship between InDels and sheep growth traits. *GHR* showed high variation in Chinese sheep, and *GHR*-53 showed the highest minimum allele frequency (MAF). There was only one InDel mutation site in both *GHRH* and *GHRHR*. The genotype frequencies of Hu sheep (HS), Tong sheep (TS), and Lanzhou fat-tail sheep (LFTS) were quite different from other breeds. The association between *GHR*, *GHRH*, and *GHRHR* InDels and body size traits in seven varieties were analyzed. The results showed that there was no significant relationship between *GHRH* and body size traits in the seven sheep populations. There was a positive association between *GHR*-21 and hip height of LFSH (*p* < 0.05). *GHR*-43 reduced body height and chest depth of Small tail han sheep (STHS) and hip width of TS. *GHR*-44 significantly affected the body weight of HS, the body height of STHS and the head depth of TS. *GHR*-53 significantly reduced cannon girth of HS, chest of STHS and forehead width of TS. *GHRHR*-2 significantly reduced the body weight of LFHS. To sum up, this study revealed the effects of *GHR*, *GHRH*, and *GHRHR* InDels on sheep phenotypic traits, which indicated their potential application prospects in the genetic improvement of mutton sheep.

## 1. Introduction

InDel is a phenomenon of vacancy caused by homologous alignment [1]. An InDel creates a length polymorphism marker and is one of the most abundant variation types in animal and plant genomes [2,3]. An InDel is defined as a variation in which an insertion or deletion is lower than 50 bp [4,5]. This sequence variation is important for disease susceptibility, phenotypic diversity, and evolutionary adaptation. It is achieved by promoting the initiation or extension of transcription, increasing the stability of mRNA in the nucleus, and enhancing gene expression [6]. As a high frequency genetic marker, InDel has been applied to population genetic analysis of animals and plants, molecular-assisted breeding, human forensic genetics, medical diagnosis, and other fields [7,8,9,10,11]. Molecular marker-assisted selection plays an important role in the process of molecular breeding of beef cattle and sheep in China [12,13,14]. With the development of InDel markers located on functional genes, some InDels have been identified as functional markers related to the growth of mutton sheep [15,16].

*GH* is one of the most important genes in the growth and development of vertebrates, and it produces effects by binding to its receptor *GHR*. It is reported that *GH* and *GHR* are important candidate genes for growth, carcass and lactation traits of domestic animals. GHRH was synthesized and secreted by the hypothalamus. For instance, *GH*, *GHRH* and its receptor *GHRHR* are the main regulators of the GH axis, which could regulate the generation and release of GH from the hypothalamus and promote the proliferation of growth cells. Excessive GHRH causes growth-promoting cell proliferation and increased GH secretion. GHRH deficiency is caused by *GHRH* mutation or inactivation, which can cause adverse symptoms such as poor growth promoting cell regeneration and single growth hormone deficiency (IGHD) [17]. *GHRH* plays a very important role in the growth and development of acquired animals. It has been reported that most mutations in *GHRH* and *GHRHR* lead to human IGHD. Similarly, mutations in *GHRH* and *GHRHR* in other mammals also affect the growth and development of animals [18]. The GH growth axis is an important endocrine and metabolic axis in animals, which regulates the growth and development of animals throughout their whole lives. In recent years, studies have found that the SNP of *GHR*, *insulin-like growth factor binding protein* (*IGFBP*), *GHRHR*, *epidermal growth factor* (*EGF*), and other genes had an impact on the growth axis of the hypothalamus GH, thus affecting the growth and development of the sheep. Gorlov et al. [19] found that InDel of *GH* was significantly associated with carcass weight, slaughter weight, slaughter yield of Salsk male lamb; Armstrong et al. [20] reported that SNP RS400358099 in *GHRHR* could regulate the growth traits of Texel lambs; Jia et al. [21] found that *GH* polymorphism was significantly correlated with growth traits of Tibetan sheep and Poll Dorset sheep; Valeh et al. [22] found that Baluchi sheep with genotype G/G of *GHR* had a superior daily Gain from Birth to Weaning (GBW). GH axis related gene InDel has also been reported to influence the growth of beef cattle, Ferraz et al. [23] identified six new polymorphisms of the bovine *GH* (one InDel and five SNPs), which could be used as molecular markers in genetic studies. Szmatoła [24] found that one of the most important detected genes was *GHR*, with a known influence on the milk and meat traits of the 11 cattle breeds maintained in Poland from the BovineSNP50 microarrays (Illumina).

The latitude and longitude span of China is large, and there are differences in breeding varieties in different regions. Therefore, we chose HS in the east, LFTS, STHS, and TS in the west, and three native breeds in Xinjiang. Among them, LFTS and TS belong to fat tail sheep, while STHS belong to small tail sheep. The seven breeds, all of which belong to mutton sheep, are well commonly farmed locally. In this study, we speculated that the InDel of *GHR*, *GHRH,* and *GHRHR* may affect the growth of seven Chinese local sheep breeds. Therefore, this study firstly investigated the distribution of seven Chinese local sheep varieties *GHR*, *GHRH*, *GHRHR*-InDel, and then carried out correlation analysis between all InDels and phenotypic traits of sheep, in order to explain the genetic effect of *GHR*, *GHRH*, *GHRHR*-InDel in Chinese local sheep varieties, so as to provide useful information for the genetic protection and improvement of Chinese sheep.

## 2. Material and Methods

### 2.1. DNA Samples and Data Collection

This research was approved by Northwest A&F University ethic committee on 20 May 2017 (ethic code: NWAFAC1019). A total of 969 sheep were used in this study. All animals were adults, healthy, and unrelated. All animals within a breed were managed in the same way, and sufficient feed was provided by total metabolic rate (TMR). Blood samples and body measurements were collected from seven breeds included STHS (*n* = 184, female), TS (*n* = 268, female), LFTS (*n* = 67, female), HS (*n* = 166, female), Duolang sheep (DLS, *n* = 92, female and male), Bashbay sheep (BBS, *n* = 96, female and male), and Altay sheep (ATS, *n* = 96, female and male), Table 1 gives sample details. The body weight (BW), body height (BH), body length (BL), chest circumference (ChC), chest depth (ChD), chest width (ChW), hucklebone width (HuW), hip width (HW), and cannon circumference (CaC) were reported using standard measurement method [25]. Consequently, body length index (BLI), chest circumference index (ChCI), chest width index (ChWI), cannon circumference index (CaCI), hucklebone width index (HuWI), and trunk index (TI) were calculated [26].

### 2.2. DNA Isolation and Genomic DNA Pools Construction

DNA was extracted from blood and musculature of ear margin using the proteinase-K-chloroform method. The DNA sample quality was tested by Nanodrop 1000 (Thermo Scientific, Waltham, MA, USA). All DNA samples were diluted to 50 ng/μL and stored at −20 °C [27]. DNA mixture was prepared by randomly selecting 20 individuals of each breed to mix DNA equally.

### 2.3. Primer Design and PCR Amplification

According to the potential InDel sites of *GH*, *GHR*, *GHRH*, and *GHRHR* published in Ensembl database, specific primers were designed with NC_040262.1, NC_040267.1, NC_040264.1, and NC_0402551.1 as reference genomes, respectively. Primer information, annealing temperature, and mutation types of mutation sites are shown in Table S1. Sites with variation base number greater than 5 bp were screened because it could not be identified by agarose gel electrophoresis when variation base number was less than 5 bp. The primers (Sangon Biotech, Co., Ltd., Shanghai, China.) were diluted to 10 ng/μL according to the instructions. The annealing temperature of the primers were determined by touch-down PCR with DNA pool as template. Each PCR was performed with 20 μL reaction, including 10 μL 2 × Taq PCR Master Mix (Sangon Biotech (Shanghai) Co., Ltd.), 1 μL genomic DNA (50 ng/μL), and 10 pmol primers. The PCR protocol was performed 5 min predegeneration, followed by 10 cycles of 95 °C at 30 s, 60 °C at 30 s (started at 60 °C and drops by 1 °C per cycle), 72 °C at 30 s, 25 cycles of 95 °C at 30 s, annealing temperature at 30 s, 72 °C at 30 s, finally extend for 10 min. The PCR products were genotyped by 3% agarose gel electrophoresis except *GHRH* for which individual genotypes were determined by Sanger sequencing. Ten samples of each genotype were randomly selected for Sanger sequencing verification at each site [14]. The primer information of the fragments identified as variants are shown in Table 2.

### 2.4. Statistical Analyses

Microsoft Excel software was used to collate all individual genotypes at each site and calculate genotype frequency and allele frequency. The Sanger Atlas sequence data and the reference genome were compared with Bioedit [28]. The online website www.Msrcall.com was used to calculate Hardy–Weinberg equilibrium (HWE), homozygosity (Ho), heterozygosity (He), effective allele numbers (Ne), and polymorphism information content (PIC), which was based on Nei’s method [29,30]. Chi-squared tests of different genotypic frequencies and breeds were performed by “χ^2^ calculator”. Linkage disequilibrium was performed by SHEsis online platform (http://analysis.bio-x.cn). Association analysis between InDel and body measurements were conducted by SPSS software (version 18.0) (IBM, Armonk, NY, USA) general linear mixed effects model [31], followed by least significant difference post hoc test, the structure of model was Y_ijk_ = μ + G_i_ (*II*, *ID* or *DD* genotype) + A_j_ + e_ijk_, while Y_ijk_ was the phenotypic observations; μ was the mean of the phenotypic observations; G_i_ was fixed effect of the ith genotype of the InDel; A_j_ was the effect of age in ith; e_ijk_ was the residual effect, *II* was insertion and insertion, *ID* was insertion and deletion, *DD* was deletion and deletion [32].

## 3. Results

### 3.1. InDel Identification and Distribution

**Figure 1:**
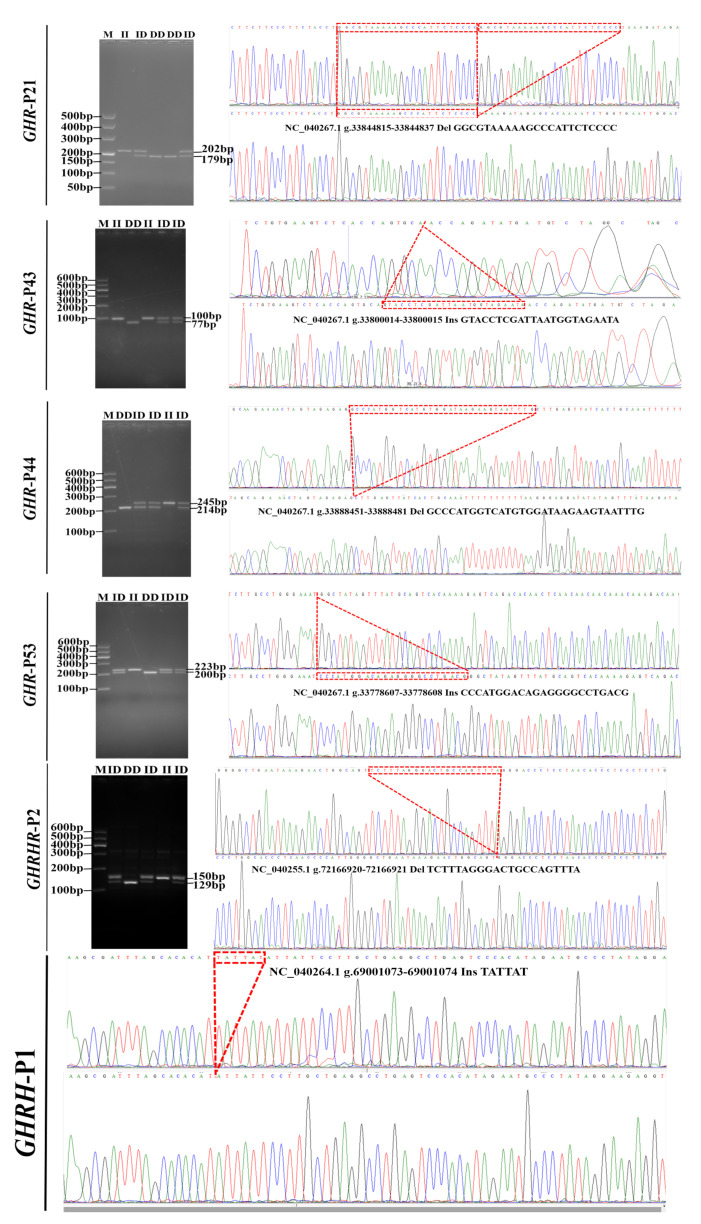
The electrophoresis diagram and sequence diagrams of six loci in *GHR*, *GHRH,* and *GHRHR.*

InDel loci were not detected for *GH*. Six InDel loci were confirmed from 62 potential loci within *GHR*, *GHRH,* and *GHRHR* by 3% gel electrophoresis and Sanger sequencing. Four InDel loci detected in *GHR* and one InDel locus within *GHRH* and *GHRHR* were identified (Table 2). Sanger sequencing revealed that 23 bases were missing at the *GHR*-21, which was located at NC_040267.1 g.33844815-33844837, and the missing bases were GGCGTAAAAAGCCCATTCTCCCC. At *GHR*-43 locus, 23 bases were inserted at NC_040267.1 g.33800014-33800015, and the inserted bases were GTACCTCGATTAATGGTAGAATA. The GCCCATGGTCATGTGGATAAGAAGTAATTTG fragment was deleted from the *GHR*-44 at NC_040267.1 g.33888451-33888481. The above three loci were all located in the upstream region of *GHR*. In addition, a 23 bp insertion mutation, *GHR*-53, was found in the first intron region of *GHR*. The *GHR*-53 was located in NC_040267.1 g.33778607-33778608, and the insertion sequence was CCCATGGACAGAGGGGCCTGACG. TATTAT fragment were inserted in *GHRH* promoter region NC_040264.1 g.69001073-69001074. The TCTTTAGGGACTGCCAGTTTA fragment were missing from the 12th intron NC_040255.1 g.72166920-7216692 of *GHRHR* (Figure 1).

### 3.2. Population Genetic Analysis of Six Mutation Sites in Seven Varieties

**Figure 2:**
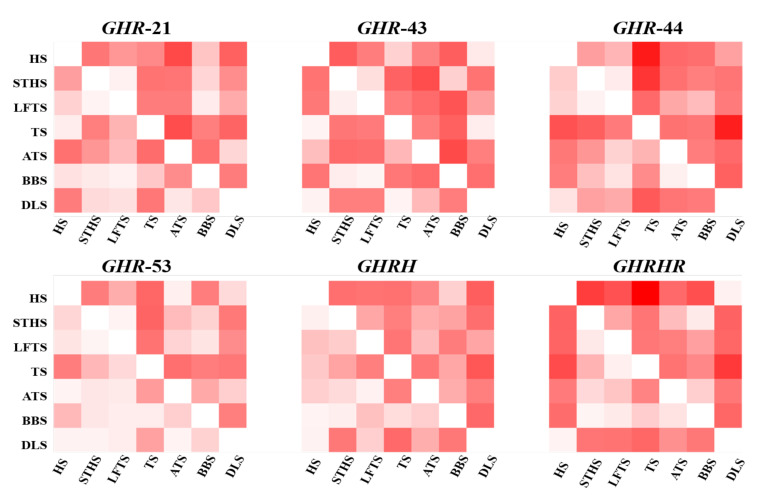
Difference analysis of InDel (insertion/deletion) distribution among populations.

Six loci of all samples were amplified by PCR and genotyping was performed by 3% agarose gel electrophoresis. The *DD* genotype at *GHR*-21, *GHR*-43, and *GHR*-44 was the dominant genotype, and the *deletion* (*D*) allele frequency was much higher than the I allele frequency. The *ID* genotype at *GHR*-53 was the dominant genotype. The *D* allele frequency was similar to the *insertion* (*I*) allele. Only TS had higher *I* allele frequency than the *D* allele. For the *GHR*-21 locus, except HS and TS population, the rest were in Hardy–Weinberg equilibrium (Table S2); for the *GHR*-44 site, the population ATS and LFTS were not in genetic balance; for the *GHR*-53 locus, except the STHS and TS population, the other groups were in Hardy–Weinberg equilibrium. Population genetic analysis revealed that the Ho and He of *GHR*-53 were close to 0.5, and the Ho of the remaining four loci was higher than the He; the Ne of *GHR*-53 was close to 2; the PIC about four loci were between 0.2 and 0.4, *GHR*-53 was moderate PIC in all the populations (0.25 < PIC < 0.5), while some populations at the other three loci achieved intermediate PIC (0 < PIC < 0.25) and others achieved moderate PIC (0.25 < PIC < 0.5). The dominant genotype of *GHRH* was *DD*, and the frequency of the *D* allele was much higher than the *I* allele. HS, ATS, BBS, and DLS were in genetic balance (*p* > 0.05), and the Ho was higher than He. As for PIC, except ATS and DLS achieving low polymorphism (0 < PIC < 0.25), the other five sheep breeds were all intermediate polymorphism (0.25 < PIC < 0.5) (Table 3). Chi-square was used to analyze the differences of genotypes and allele distributions among populations. The distribution of alleles and genotypes of HS, TS, and DLS were significantly different from other populations (*p* < 0.05) (Figure 2).

### 3.3. InDel Association Analyses

**Figure 3:**
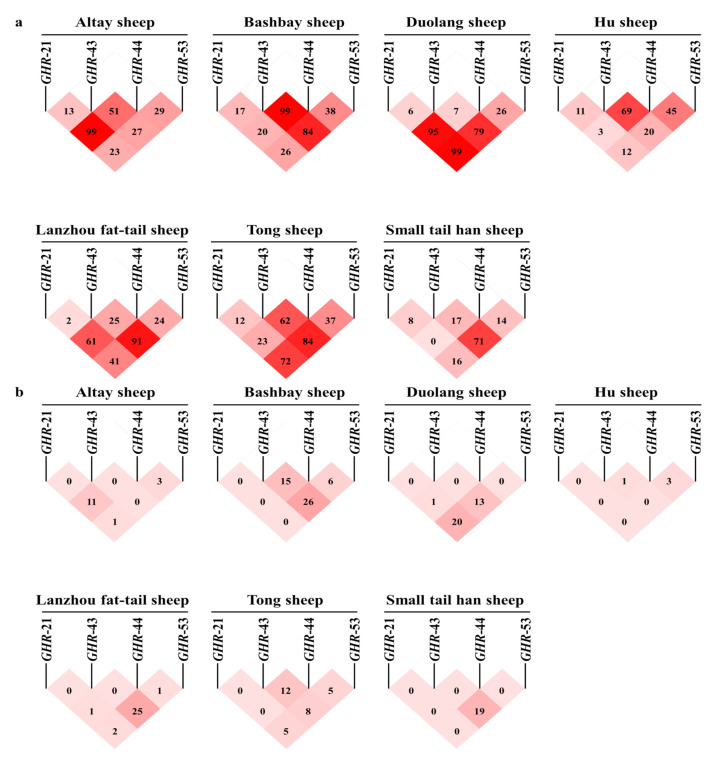
Linkage analysis of four variation sites of *GHR* in seven populations. (**a**) D’ analysis of seven sheep breeds in four GHR loci; (**b**) r2 analysis of seven sheep breeds in four GHR loci.

Linkage analysis of the four *GHR* mutation sites revealed that the four sites were not linked (strong linkage was indicated when D’ > 0.95 or r^2^ > 0.33), as shown in Figure 3. Therefore, only the relationship between a single mutation site and growth traits was analyzed, without haplotype analysis. Table 4 lists all the significant associations between the variation at each locus and the production traits of interest. It was found that hip height in the *ID* genotype of LFHS population was significantly reduced on account of *GHR*-21 (*p* < 0.05), and *DD* genotype was beneficial for LFHS. *GHR*-43 mutation significantly reduced the body height and chest depth of the STHS population (*p* < 0.05), but significantly increased hip width in the TS population (*p* < 0.01), *DD* genotype was beneficial for STHS and HS. For *GHR*-44, the body weight of the HS population and the STHS population changed in exactly the opposite direction due to the presence of fragment deletion (*p* < 0.05). At the same time, the head depth of TS group is reduced (*p* < 0.05). Mutations at *GHR*-53 reduced cannon girth and chest of the HS population and forehead width of TS population (*p* < 0.05), *DD* genotype was beneficial for STHS and HS. *DD* genotype of *GHRHR* mutation resulted in significant decrease of body weight of LFHS population (*p* < 0.05). The *GHRH* mutation was not significantly associated with sheep growth traits (Table S3).

## 4. Discussion

InDel variation could affect animal growth traits, which has been reported in many studies, for example, InDel on *FTO* were associated with tail length and growth trait in the Tong sheep [33]. A 4 bp InDel in *Sox9* 3′ UTR was significantly correlated with goat body length, heart girth, and hip width. *Sox9*, *HIAT1*, *MSTN*, and *CSN1S1* strongly effect growth traits in goats [29,34,35]. There have been many reports on the effects of the GH axis related gene mutation on animal growth. The heterozygous haplotype C261G/G263C, in exon 21 of the *IGF1R* was found that associated with the average daily gain during the early stages of life (from birth to six moon of age) and could be used as a genetic marker for selection of growth traits in Egyptian buffalo [36]. Results of Mullen et al. [37] demonstrated the multifaceted influences of *IGF-1* on milk production and growth traits in cattle. However, there are few studies on the relationship between InDel and mutton sheep growth.

In this study, InDel were investigated as molecule markers that might be associated with important economic traits and therefore potentially useful in marker-assisted breeding programmes. Three of them were located in *GHR*-5′ UTR, and the other was located in the first intron region of *GHR*. GH is a macromolecular substance, which cannot directly pass through the cell membrane, but must activate the signal transduction pathway by binding to GHR to transmit the information into the cell, so that GH can play its biological function. GH combines with GHR, catalyzes GHR dimerization, and activates GHR. GHR dimerization exposes the binding sites of receptors and ligands, increasing the affinity between receptors and ligands, and ensuring the smooth progress of downstream signal transduction. *GHR* has multiple first exons to choose from, so there are also multiple 5 ’UTRs, which also leads to the molecular diversity of *GHR*. GHR includes the extracellular region, transmembrane region, and intracellular region. The receptor and ligand-binding regions are located in two conserved sequences in the intracellular region, so the intracellular region plays a crucial role in the binding of GH. GHRH is a polypeptide secreted by the hypothalamus. As a positive regulator of GH, GHRH mainly promotes the synthesis and secretion of GH by the pituitary gland. GHRHR, as the GHRH receptor, binds to GHRH and promotes the release of GH by increasing intracellular cAMP and Ca^2+^, thus promoting the growth and development of the body. *GH*, *GHR*, *GHRH,* and *GHRHR* are the most critical genes in the GH axis, their diversity plays an important role in the growth regulation of animals. Maj et al. [38] studied four SNPs of *GHR* 5’UTR and the meat production performance of Holstein cattle in Poland, and found that a single genotype had no effect on the production performance. Combined genotype analysis found that polymorphism had a significant effect on feed utilization and carcass weight of cattle. Zhang et al. [39] used PCR-SSCP to detect the polymorphism of *GHR* 3’UTR regulation region in 392 Nanjiang yellow sheep and 49 Boer goats, and carried out correlation analysis with the body weight traits. According to the least-squares analysis, the genotype effect in the two goat populations had no significant effect on the initial birth weight, but had a significant effect on the first-year body weight. Human IGHD is a family genetic disease with *GHRHR* mutation leading to GH deficiency. Seventeen mutations have been reported in *GHRHR*, and these mutations will lead to severe growth disorders in patients [40]. At present, there are few studies on the association between SNP of *GH*, *GHR,* and *GHRHR* with sheep growth traits, and only one breed of sheep was tested and analyzed [18,19,20,21,22,41,42]. The association between InDel of *GH* and sheep growth has only been reported in Luxi Blackhead sheep [15]. For the first time, this study examined the distribution of the InDel loci of the GH axis key genes in multiple breeds of Chinese sheep and the association analysis with different population growth traits. In this study, we found that *GHR* and *GHRHR* were associated with growth traits of four sheep breeds. *GHR*-InDel had a great effect on STHS and TS product traits, which could affect body height, chest depth of STHS, chest, hip width, head depth, and forehead width of TS, and could also regulate STHS body height, chest and LFHS hip height. In conclusion, *GHR*-InDel may regulate the important growth indicators of four sheep breeds, and the selection of dominant genotypes can be used as the theoretical basis for molecular breeding about improving the production traits and meat yield of the four sheep breeds. *GHGH*-InDel had almost no association with the growth of Chinese sheep, and *GHRHR*-InDel had a strong correlation with the weight of LFHS, the *DD* genotype group gained 33% more weight than the *II* genotype group. A growing number of studies have demonstrated that intron regions and 5′ UTR regions of genes play an important role in regulating gene expression levels [43]. Introns can regulate gene transcription rate, gene transcription length, gene structure, and provide binding sites for binding proteins. For example, Lu et al. [44] found that the human *β-Globin* is a highly intron-dependent gene, and the amount of its mature mRNA and the utilization rate of translation decreased significantly in the absence of splicing. Chang et al. [45] found that there was an important regulatory element in pig *MyHC* introns that regulated the initiation of transcription and enhanced gene expression. Introns have positional effects and directional dependence in gene expression [6]. We hypothesized that this mutation caused the structure and function of *GHRHR*, which further affected the binding of *GHRHR* and *GHRH* or the sensitivity of *GHRH* to activate cAMP.

## 5. Conclusions

In summary, we identified six InDels from 62 potential loci within the GH axis in 969 individuals from seven Chinese sheep populations, including four InDels in *GHR*, one InDel in *GHRH,* and one InDel in *GHRHR*. InDel in *GHRHR* was associated with LFHS production trait, and three mutations of *GHR* were strongly associated with HS, TS, LFHS, and STHS growth traits. Our findings implied that *GHR*-InDel could be used as a promising marker for beef sheep breeding by selecting the advantageous genotype.

## Figures and Tables

**Table 1:** Information of seven sheep breeds in this study.

Breed	Abbreviation	Sampling Location	*n*	Sample Form
Small tail han	STHS	Lanzhou city (Gansu province)	184	Musculature of ear margin
Tong	TS	Baishui county (Shanxi province)	268	Venous blood
Lanzhou fat-tail	LFTS	Lanzhou city (Gansu province)	67	Musculature of ear margin
Hu	HS	Huzhou city (Zhejiang province)	166	Venous blood
Duolang	DLS	Shihezi city (Xinjiang autonomous region)	92	Venous blood
Bashbay	BBS	Shihezi city (Xinjiang autonomous region)	96	Venous blood
Altay	ATS	Altay city (Xinjiang autonomous region)	96	Venous blood
Total			969	

Notes: *n*—sample size.

**Table 2:** PCR primer sequences of sheep *GHR*, *GHRH*, and *GHRHR.*

Gene(Gene ID)	Name	Primer Sequences (5′-3′)	Product Size (bp)	Notes
*GHR* (443333)	*GHR*-21	F: CGGTCCAATTCACCAGAT	202 − 23	Upstream
		R: AGAATCCCACGGACAAAG		
	*GHR*-43	F: CTGTGAAGTCTCACCAGTGC	77 + 23	Upstream
		R: GGATAGGCAGAATGCTAAAG		
	*GHR*-44	F: GAAATACCCTTGTGGACAGA	245 − 31	Upstream
		R: CTGGATTTATTGTTATTGTCTATTG		
	*GHR*-53	F: ATGTTAGGCAGCCAAAAGG	200 + 23	1st intron
		R: GCCCAACCCAATGTTAATAGA		
*GHRH* (100101237)	*GHRH*	F: ACGACTGAAGCGATTTAGCAC	159 + 6	Promoter region
		R: TCCATCTGTAAAATGGGCATG		
*GHRHR* (443511)	*GHRHR*-2	F: AACCCCTGTCTCAGTTTCTCC	129 + 21	12th intron
		R: GATCTCAGTCCTCACCTCCAA		

Notes: *GH*: growth hormone; *GHR*: growth hormone receptor; *GHRH*: growth hormone releasing hormone; *GHRHR*: growth hormone releasing hormone receptor; F: forward primer; R: reverse primer; −: deletion; +: insertion.

**Table 3:** Genotypes, alleles, He, Ne, and PIC for InDels of the sheep *GHR*, *GHRH,* and *GHRHR.*

Locus	Breed	Size	Genotypic Frequency			Allelic Frequency		Population Parameters			
		*n*	*II*	*ID*	*DD*	*I*	*D*	Ho	He	Ne	PIC
*GHR*-21	HS	184	161	18	5	0.924	0.076	0.859	0.141	1.164	0.131
	STHS	268	203	57	8	0.864	0.136	0.765	0.235	1.308	0.208
	LFTS	67	52	13	2	0.873	0.127	0.778	0.222	1.285	0.197
	TS	166	146	20	0	0.940	0.060	0.887	0.113	1.128	0.107
	ATS	92	53	36	3	0.772	0.228	0.648	0.352	1.544	0.290
	BBS	96	78	15	3	0.891	0.109	0.805	0.195	1.242	0.176
	DLS	96	63	31	2	0.818	0.182	0.702	0.298	1.425	0.254
*GHR*-43	HS	184	132	47	5	0.845	0.155	0.738	0.262	1.355	0.228
	STHS	268	133	111	24	0.703	0.297	0.583	0.417	1.716	0.330
	LFTS	67	37	16	4	0.672	0.328	0.621	0.389	1.610	0.307
	TS	165	114	49	2	0.839	0.161	0.730	0.270	1.369	0.233
	ATS	92	76	16	0	0.913	0.087	0.841	0.159	1.189	0.146
	BBS	96	41	47	8	0.672	0.328	0.559	0.441	1.789	0.344
	DLS	96	66	28	2	0.833	0.167	0.722	0.278	1.385	0.239
*GHR*-44	HS	184	142	38	4	0.875	0.125	0.781	0.219	1.280	0.377
	STHS	268	184	76	8	0.828	0.172	0.716	0.284	1.397	0.244
	LFTS	67	45	19	3	0.813	0.187	0.696	0.304	1.436	0.257
	TS	166	62	83	21	0.623	0.377	0.531	0.469	1.885	0.359
	ATS	92	54	26	12	0.728	0.272	0.604	0.396	1.655	0.317
	BBS	96	53	39	4	0.755	0.245	0.630	0.370	1.587	0.301
	DLS	96	82	11	3	0.911	0.089	0.839	0.161	1.192	0.148
*GHR*-53	HS	184	49	97	38	0.530	0.470	0.502	0.498	1.993	0.374
	STHS	268	70	114	84	0.474	0.526	0.501	0.499	1.994	0.374
	LFTS	67	17	29	21	0.470	0.530	0.502	0.498	1.993	0.374
	TS	166	16	98	52	0.392	0.608	0.524	0.476	1.910	0.363
	ATS	92	25	46	21	0.522	0.478	0.501	0.499	1.996	0.375
	BBS	96	19	44	33	0.427	0.573	0.511	0.489	1.958	0.301
	DLS	96	21	57	18	0.516	0.484	0.500	0.500	1.998	0.374
*GHRH*	HS	184	6	73	105	0.231	0.769	0.645	0.355	1.550	0.292
	STHS	268	24	68	176	0.216	0.784	0.661	0.339	1.513	0.282
	LFTS	67	4	11	52	0.142	0.858	0.757	0.243	1.322	0.408
	TS	166	23	53	90	0.298	0.702	0.581	0.419	1.720	0.331
	ATS	92	3	24	65	0.163	0.837	0.727	0.273	1.375	0.236
	BBS	96	6	34	56	0.240	0.760	0.636	0.364	1.573	0.298
	DLS	96	1	13	82	0.078	0.922	0.856	0.144	1.168	0.134
*GHRHR*-2	HS	184	167	15	2	0.948	0.052	0.902	0.097	1.109	0.093
	STHS	268	173	80	15	0.795	0.205	0.673	0.326	1.484	0.273
	LFTS	67	42	17	8	0.754	0.246	0.502	0.498	1.993	0.374
	TS	166	82	76	8	0.723	0.277	0.629	0.371	1.590	0.302
	ATS	92	66	24	2	0.848	0.152	0.742	0.258	1.348	0.225
	BBS	96	61	31	4	0.797	0.203	0.676	0.324	1.489	0.271
	DLS	96	86	9	1	0.943	0.057	0.892	0.108	1.121	0.102

Notes: *GHR*: *growth hormone receptor*; *GHRH: growth hormone releasing hormone; GHRHR: growth hormone releasing hormone receptor*; Ho: homozygosity; He: heterozygosity; Ne: effective allele numbers; PIC: polymorphism information content; STHS: Small tail han sheep; TS: Tong sheep; LFTS: Lanzhou fat-tail sheep: HS: Hu sheep; DLS: Duolang sheep; BBS: Bashbay sheep; ATS: Altay sheep; *II*: *insertion/insertion*; *ID*: *insertion/deletion*; *DD*: *deletion/deletion*.

**Table 4:** Relationship between variations of *GHR*, *GHRH,* and *GHRHR* and their growth traits.

Loci	Breeds	Growth Traits	Observed Genotypes (LSM ^a^ ± SE)			*p* Values
			*II*	*ID*	*DD*	
*GHR*-21	LFHS	Hip height (cm)	77.92 ^a^ ± 1.27 (*n* = 25)	71.50 ^b^ ± 2.07 (*n* = 7)	84.50 ^a^ ± 3.50 (*n* = 2)	0.019
*GHR*-43	STHS	Body height (cm)	62.60 ^b^ ± 0.45 (*n* = 90)	64.26 ^a^ ± 0.46 (*n* = 80)	62.37 ^a,b^ ± 0.85 (*n* = 20)	0.023
	STHS	Chest depth (cm)	27.17 ^b^ ± 0.29 (*n* = 90)	27.89 ^a,b^ ± 0.27 (*n* = 80)	28.66 ^a^ ± 0.61 (*n* = 20)	0.036
	TS	Hip width (cm)	13.88 ^b^ ± 0.27 (*n* = 48)	15.10 ^a,b^ ± 0.30 (*n* = 27)	16.50 ^a^ ± 0.50 (*n* = 2)	0.005
*GHR*-44	HS	Body weight (kg)	32.73 ^a^ ± 0.36 (*n* = 141)	31.03 ^b^ ± 0.83 (*n* = 38)	27.18 ^b^ ± 1.94 (*n* = 4)	0.009
	STHS	Body height (cm)	62.82 ^b^ ± 0.36 (*n* = 129)	64.00 ^a,b^ ± 0.62 (*n* = 54)	66.14 ^a^ ± 1.30 (*n* = 7)	0.044
	TS	Head depth (cm)	14.53 ^b^ ± 1.32 (*n* = 26)	14.94 ^a^ ± 0.11 (*n* = 42)	14.20 ^b^ ± 0.32 (*n* = 10)	0.007
*GHR*-53	HS	Cannon girth (cm)	6.96 ^b^ ± 0.09 (*n* = 49)	7.12 ^a,b^ ± 0.05 (*n* = 97)	7.25 ^a^ ± 0.10 (*n* = 38)	0.049
	STHS	Chest circumference (cm)	72.20 ^a,b^ ± 0.78 (*n* = 52)	71.00 ^b^ ± 0.61 (*n* = 83)	73.60 ^a^ ± 0.91 (*n* = 55)	0.043
	TS	Forehead width (cm)	12.00 ^b^ ± 0.27 (*n* = 5)	12.86 ^a^ ± 0.11 (*n* = 47)	12.90 ^a^ ± 0.15 (*n* = 25)	0.049
*GHRHR*-2	LFHS	Body weight (kg)	47.27 ^b^ ± 2.96 (*n* = 23)	58.95 ^a,b^ ± 4.88 (*n* = 8)	62.87 ^a^ ± 3.68 (*n* = 3)	0.050

Notes: STHS: Small tail han sheep; TS: Tong sheep; LFTS: Lanzhou fat-tail sheep: HS: Hu sheep; LSM: least squares technique; SE: standard error; STHS: Small tail han sheep; TS: Tong sheep; LFTS: Lanzhou fat-tail sheep: HS: Hu sheep; *II*: *insertion/insertion*; *ID*: *insertion/deletion*; *DD*: *deletion/deletion*. ^a,b^ Values with different superscript letters in the same row differ at *p* < 0.05 for lower-case.

## Supplementary Materials

The following are available online at https://www.mdpi.com/2076-2615/10/10/1883/s1, Table S1: PCR primer sequences of InDels in sheep *GH*, *GHR*, *GHRH,* and *GHRHR* genes, Table S2: Hardy–Weinberg equilibrium test for gene frequency and genotype frequency of six mutant sites in seven populations, Table S3: Relationship between variations of *GHR*, *GHRH,* and *GHRHR* and their growth traits (*p* < 0.1).
