# Supplementary material for: Novel InDels of GHR, GHRH, GHRHR and Their Association with Growth Traits in Seven Chinese Sheep Breeds
Source: Animals (Basel). 2020 Oct 15;10(10):1883. doi: 10.3390/ani10101883 (PMC7602648; doi:10.3390/ani10101883)
Supplement: Supplementary file 1 [file animals-10-01883-s001.pdf]

Supplementary Material: Novel InDels of *GHR*, *GHRH*, *GHRHR* and Their Association with Growth Traits in Seven Chinese Sheep Breeds

Mingli Wu, Haidong Zhao, Xiaoqin Tang, Qi Li, Xiaohua Yi, Shirong Liu and Xiuzhu Sun

Table S1. PCR primer sequences of InDels in sheep *GH, GHR, GHRH* and *GHRHR* gene.

Gene	Name	Primer Sequences (5′-3′)		Tm (℃)	Products(bp)
GH	*GH*-P1	F: CTTGACCTGCATACAGATTT	R: AAAAGGCAGAGGAACCAC	TD-PCR	206 + 7
	GHR-P1	F: TTAGTCCAAATGCCAAAG	R: TAAGTTGTCCTTCCACCC	TD-PCR	231 − 16
	GHR-P2	F: AAACGTGGAGAAGGGACT	R: TTAATCGGAGGATAACTGC	TD-PCR	295 − 15
	GHR-P3	F: CGACCTAACCTGCCCTGTG	R: TGACTCTGCGACCCTTTG	TD-PCR	278 − 6
	GHR-P4	F: GGGATTCCCTTCACTTCTCA	R: AGGCTCCTTGTCTTTCTCGT	TD-PCR	296 − 12
	GHR-P5	F: GGATACCTGGGTTCAATC	R: GGTAACCTCCTTTGTTTCT	TD-PCR	191 + 6
	GHR-P6	F: TTTGCTCAGGAAGGTTGG	R: TCAGCTTAAACATGGAAGAC	TD-PCR	196 − 11
	GHR-P7	F: GGGAAACCCCACATAGTA	R: TGCATATTAGAAATGTAGCC	TD-PCR	217 − 7
	GHR-P8	F: TCTTGTTTCCTACCCACCAC	R: TGCATGGGCCATCAGTTT	TD-PCR	168 + 7
	GHR-P9	F: TGGCAGGAAAGCTATGAC	R: TCAGTCCTTCCAATGAGTAT	TD-PCR	259 + 11
	GHR-P10	F: CTACCAAAGGCTGGATTC	R: TTTCAGGGGCTTTAAGTT	TD-PCR	216 − 7
	GHR-P11	F: AAGAAATAGTTTGGATACG	R: ACATTGACCAAAGCATTA	TD-PCR	180 + 6
	GHR-P12	F: TTAGTTTTGCCTCATCAT	R: GGGTAGAATCAGCATCTT	TD-PCR	297 + 19
	GHR-P13	F: TTCCAAGGAGCAAGCATC	R: GGCTGGCTTAAAACTCAA	TD-PCR	174 − 14
GHR	*GHR*-P14	F: CTAAAGCCTTTGATTGTG	R: AATAAGCAGGGTGACAAT	TD-PCR	193 − 13
	GHR-P15	F: GCAACAACACTTCCCTGAC	R: TTAGGTCATGCCTGAAAGG	TD-PCR	276 + 7
	GHR-P16	F: TCCCACAGCCGTCTACTC	R: TCTGGGGATATGTCAAGGTC	TD-PCR	257 − 10
	GHR-P17	F: TCAGGCTCTGGGAGAAAG	R: CCAAGGAAATTAGCGACAT	TD-PCR	263 + 9
	GHR-P18	F: TGGGCCTCGCTGAGTATA	R: ACCCAAAAGACAGACAAACATA	TD-PCR	168 − 6
	GHR-P19	F: ATAGATTGAAAGCCCTGGTT	R: CTGGGAGTTGGTGATGGA	TD-PCR	229 − 11
	GHR-P20	F: CAGGCTTCCTTGTCTTTCA	R: AGCTTCGATACTTTGGTCAC	TD-PCR	284 − 14
	GHR-P21	F: CGGTCCAATTCACCAGAT	R: AGAATCCCACGGACAAAG	TD-PCR	202 − 23
	GHR-P22	F: CGTGCCTTACCTGCTTCC	R: AGTCACTCTGTCCTCTGTGGG	TD-PCR	204 − 14
	GHR-P23	F: TTTTCCCTCTTCACTTCT	R: CTCTTCATTCATAACAGCA	TD-PCR	288 − 13
	GHR-P24	F: AATAAGCAAAGCTAACAATCC	R: AAACAGCACGCTGTCAAG	TD-PCR	176 − 18
	GHR-P25	F: CAGTGACCAAAACCATCC	R: TTATCTCACCTTGCTATTCC	TD-PCR	185 − 8
	GHR-P26	F: TTGGAGATACAGCAATGA	R: GCTCTTTCCTGGTAACTT	TD-PCR	162 + 19
	GHR-P27	F: GTGGACACCAGGTGAGAAA	R: GAAAGAGGAAGAAAAGGAAAC	TD-PCR	190 − 9

	GHR-P28	F: ATTCACTTCTTGGGTCATTT	R: GCACTTCACTTCCCTTCA	TD-PCR	188 + 6
	GHR-P29	F: GAGACCAAACCTGTCAATCC	R: CAACCATCCCATCCTCAG	TD-PCR	183 − 6
	GHR-P30	F: GGGATGCCAGAGTTTCAG	R: GATGCTGCTTCTGGTGAC	TD-PCR	253 + 7
	GHR-P31	F: AACATTTCTTCAGGAGGC	R: CTCATTTTCATGCATTGG	TD-PCR	199 − 7
	GHR-P32	F: TGACTGAGCGACTAAGCAC	R: AGTAAAGGAAGCCAGCAAT	TD-PCR	180 − 21
	GHR-P33	F: CTGAGGGCAGTGGTTGAA	R: CTCAGCAATGGCATCTTTA	TD-PCR	185 − 38
	GHR-P34	F: GCATCTGGTCCCATCATTTC	R: GCTTTCCTTATGGTCCAACTCT	TD-PCR	281 − 12
	GHR-P35	F: CGCCAGTCTCCTCTGTCCAT	R: ATAAGCTCGCCGACCTAACC	TD-PCR	260 + 46
	GHR-P36	F: AGGGTGAACTCAAGGAAAGC	R: ATAAGACCCAATGGCAAGAAC	TD-PCR	224 − 27
	GHR-P37	F: GGCTACAGTCCGTAGGGTCG	R: GGTGTTTGCTGGCAGGTTT	TD-PCR	291 − 7
	GHR-P38	F: AGGCTCCTTGTCTTTCTCGT	R: TGTGGGATTCCCTTCACTTC	TD-PCR	299 − 10
	GHR-P39	F: TCACAGTTCTAGCCAATGCTG	R: TCCGACTCTTTGCGACCC	TD-PCR	274 − 13
	GHR-P40	F: ACGCCACAGCCTCTTCTATC	R: CAAGCCCTATGTGCATTTACT	TD-PCR	238 − 16
	GHR-P41	F: CCCCTCTACCTTCCTTCTG	R: TTGGTCTTCCCTCATCTTTAT	TD-PCR	193 − 13
	GHR-P42	F: CCTGGAAATAAGAGTTTAGGA	R: AGGATGGGAGACAGTGATG	TD-PCR	286 − 19
	GHR-P43	F: CTGTGAAGTCTCACCAGTGC	R: GGATAGGCAGAATGCTAAAG	TD-PCR	100 − 23
	GHR-P44	F: GAAATACCCTTGTGGACAGA	R: CTGGATTTATTGTTATTGTCTATTG	TD-PCR	297 − 31
	GHR-P45	F: TCAGAGTCAAATCCTACCCTTGG	R: AGGATGCTTGGGGCTGTTG	TD-PCR	293 − 36
	GHR-P46	F: AAAGGCAGTTCAGGATGGG	R: AACCTTGATTGTGAATATCTAGGTAA	TD-PCR	207 − 8
	GHR-P47	F: GTGGGTTGCTGTTTCTTCC	R: ATCTGCTTACATGGTGGTATTT	TD-PCR	283 − 34
	GHR-P48	F: TTGCCTTGAAACAGAAAAC	R: AAAGAAAACAGGGGACACT	TD-PCR	221 − 18
	GHR-P49	F: GTCTACAAGGCTCCTCCATCTAC	R: GGCATCCCAGTGACTATTTCC	TD-PCR	217 − 7
	GHR-P50	F: CCGACCTAACCTGCCCTGTG	R: TGTCTGACTCTGCGACCCTTTG	TD-PCR	283 − 45
	GHR-P51	F: GCAGAGGGCAACCAGAGTA	R: GGAATGGCACAGGATCTCA	TD-PCR	258 − 15
	GHR-P52	F: GAACCAGCCAGGAAGGGAG	R: GAAGGCGATTCAGAAAGGAGAT	TD-PCR	239 − 13
	GHR-P53	F: GGGAAGCCACCAGGATTCTT	R: TACCTTGTCTTTGTTTGTTGTTGTTG	TD-PCR	120 − 23
	GHR-P54	F: AAGTATTTGTTGAATCACCAGT	R: GGCTCTATAGCTTCTTTACTGT	TD-PCR	263 − 47
	GHR-P55	F: TGCGTTCAACTGATTTGGT	R: GGTCCTTTGGTGGCCCTTA	TD-PCR	250 + 36
	GHR-P56	F: AGGAAATTTGCAGCAAGTGC	R: CACTGGCTGCTGCTTGGAAG	TD-PCR	190 − 18
	GHR-P57	F: GCTTGAGTTATCACTGCAAAT	R: CTGGATTTATTGTTATTGTCTATTG	TD-PCR	192 − 50
GHRH	*GHRH*-P1	F: GGCTACAGTCCATAGGGTCAC	R: CACCTGGGTTCAAGTTCTGC	TD-PCR	250 − 6
	GHRHR-P1	F: TCTGGTGGCCTGGCTTGGA	R: GGGATCGTGAGGACTGGGAG	TD-PCR	286 − 8
GHRHR	*GHRHR*-P2	F: AACCCCTGTCTCAGTTTCTCC	R: GATCTCAGTCCTCACCTCCAA	TD-PCR	150 − 21
	GHRHR-P3	F: CCTTGTCACCTGGAGACTTGGA	R: GAAACCCCTACCTTGCCCAC	TD-PCR	251 − 39

Notes: TD-PCR: Touch-down polymerase chain reaction; *GH*: Growth hormone; *GHR*: growth hormone receptor; *GHRH*: Growth hormone releasing hormone; *GHRHR*: Growth hormone releasing hormone receptor; F: Forward primer; R: Reverse primer; T_m: Melting temperature.

Table S2. Hardy Weinberg equilibrium test for gene frequency and genotype frequency of six mutant sites in seven populations.

Loci	HS	STHS	LFTS	TS	ATS	BBS	DLS
***GHR*-21**	0.000	0.116	0.309	0.002	0.288	0.052	0.415
***GHR*-43**	0.742	0.903	0.840	0.193	0.361	0.280	0.624
***GHR*-44**	0.448	0.964	0.591	0.403	0.006	0.333	0.004
***GHR*-53**	0.430	0.016	0.283	0.002	0.986	0.543	0.645
GHRH	0.113	0.000	0.008	0.002	0.672	0.784	0.557
***GHRHR*-2**	0.023	0.164	0.283	0.010	0.916	0.980	0.196

Table S3. Relationship between variations of *GHR*, *GHRH* and *GHRHR* and their growth traits ($p < 0.1$).

Loci	Breeds	Growth Traits	Observed Genotypes (LSM [a] ± SE)			
			II	*ID*	*DD*	***p* values**
***GHR*-21**	**HS**	thurl width	17.43 [a] ± 0.09 (*n* = 161)	17.11 [a] ± 0.31 (*n* = 18)	18.40 [a] ± 0.29 (*n* = 5)	0.075
	**STHS**	hip height	63.27 [a] ± 0.33 (*n* = 144)	61.55 [a] ± 0.76 (*n* = 42)	63.50 [a] ± 2.99 (*n* = 4)	0.069
	**LFHS**	hip height	77.92 [a] ± 1.27 (*n* = 25)	71.50 [b] ± 2.07 (*n* = 7)	84.50 [a] ± 3.50 (*n* = 2)	0.019
***GHR*-43**	**STHS**	body length	58.08 [a] ± 0.62 (*n* = 90)	60.19 [a] ± 0.75(*n* = 80)	58.84 [a] ± 1.29(*n* =2 0)	0.088
	**STHS**	body height	62.60 [b] ± 0.45 (*n* = 90)	64.26 [a] ± 0.46 (*n* = 80)	62.37 [a,b] ± 0.85 (*n* = 20)	0.023
	**STHS**	chest depth	27.17 [b] ± 0.29 (*n* = 90)	27.89 [a,b] ± 0.27 (*n* = 80)	28.66 [a] ± 0.61 (*n* = 20)	0.036
	**TS**	hip width	13.88 [b] ± 0.27 (*n* = 48)	15.10 [a,b] ± 0.30 (*n* = 27)	16.50 [a] ± 0.50 (*n* = 2)	0.005
	**TS**	tail width	18.86 [a] ± 0.45 (*n* = 48)	19.65 [a] ± 0.63 (*n* = 27)	24.25 [a] ± 0.75 (*n* = 2)	0.053

GHR-44	**HS**	chest circumference	77.14 [a] ± 0.37 (*n* = 142)	76.05a [a] ± 0.79 (*n* = 38)	72.50 [a] ± 2.36 (*n* = 4)	0.069
	**HS**	cannon girth	7.15 [a] ± 0.04 (*n* = 142)	6.97 [a] ± 0.11 (*n* = 38)	6.75 [a] ± 0.43 (*n* = 4)	0.095
	**HS**	body weight	32.73 [a] ± 0.36 (*n* = 141)	31.03 [b] ± 0.83 (*n* = 38)	27.18 [b] ± 1.94 (*n* = 4)	0.009
	**STHS**	body height	62.82 [b] ± 0.36 (*n* = 129)	64.00 [a,b] ± 0.62 (*n* = 54)	66.14 [a] ± 1.30 (*n* = 7)	0.044
	**TS**	head depth	14.53 [b] ± 1.32 (*n* = 26)	14.94 [a] ± 0.11 (*n* =4 2)	14.20 [b] ± 0.32 (*n* = 10)	0.007
GHR-53	**HS**	cannon girth	6.96 [b] ± 0.09 (*n* = 49)	7.12 [a,b] ± 0.05 (*n* = 97)	7.25 [a] ± 0.10 (*n* = 38)	0.049
	**STHS**	chest depth	27.23 [a] ± 0.33 (*n* = 52)	27.41 [a] ± 0.29 (*n* = 83)	28.33 [a] ± 0.37 (*n* = 55)	0.056
	**STHS**	chest circumference	72.20 [a,b] ± 0.78 (*n* = 52)	71.00 [b] ± 0.61 (*n* = 83)	73.60 [a] ± 0.91 (*n* = 55)	0.043
	**TS**	forehead width	12.00 [b] ± 0.27 (*n* = 5)	12.86 [a] ± 0.11 (*n* = 47)	12.90 [a] ± 0.15 (*n* = 25)	0.049
	**TS**	tail length	30.50 [a] ± 1.02 (*n* = 4)	30.53 [a] ± 0.83 (*n* = 47)	33.67 [a] ± 0.90 (*n* = 26)	0.082
GHRH	**STHS**	cannon girth	7.02 [a] ± 0.15 (*n* = 18)	6.88 [a] ± 0.13 (*n* = 40)	7.18 [a] ± 0.06 (*n* = 132)	0.061
	**TS**	hip width	48.10 [a] ± 0.69 (*n* = 10)	49.70 [a] ± 0.47 (*n* = 23)	50.23 [a] ± 0.40 (*n* = 45)	0.054
GHRHR-2	**STHS**	thurl width	62.53 [b] ± 0.41 (*n* = 120)	63.07 [a,b] ± 0.54 (*n* = 57)	65.50 [a] ± 0.54 (*n* = 13)	0.054
	**TS**	hip width	49.30 [a] ± 0.37 (*n* = 35)	50.36 [a] ± 0.42 (*n* = 41)	47.00 [a] ± 2.00 (*n* = 2)	0.055
	**LFTS**	body length	72.23 [a] ± 1.48 (*n* = 23)	76.75 [a] ± 1.87 (*n* = 8)	79.50 [a] ± 1.50 (*n* = 3)	0.089

**LFHS**	body weight	47.27 ^{b} ± 2.96 (*n* = 23)	58.95 ^{a,b} ± 4.88 (*n* = 8)	62.87 ^{a} ± 3.68 (*n* = 3)	0.050

a,b Values with different superscript letters in the same row differ at $p < 0.05$ for lower-case.

© 2020 by the authors. Licensee MDPI, Basel, Switzerland. This article is an open access article distributed under the terms and conditions of the Creative Commons Attribution (CC BY) license (http://creativecommons.org/licenses/by/4.0/).
